# The Inorganic Constituents of Vegetable Food

**Published:** 1898-08-20

**Authors:** 


### The Inorganic Constituents of Vegetable Food.

Both in this country and abroad considerable attention has been paid during the last few years to the action of the inorganic elements contained in various forms of animal and vegetable food; and Dr. Luff, who entered upon a partial consideration of this subject in his Goulstonian Lectures last year, has lately read before the Royal Medical and Chirurgical Society the further conclusions to which he has been led by a series of experiments. He started with the belief that it is practically immaterial, as far as the production of gout is concerned, whether the uric acid present in excess has been yielded by animal or by vegetable proteids, so that the same harm may result from an excessive consumption of either form; and he demonstrated that different articles of diet produce widely different effects upon the precipitation of sodium biurate, and on the solution of uratic deposits. The object of the experiments last undertaken was to ascertain the relative influences exerted by the mineral constituents of various vegetables on the solubility of sodium biurate at the temperature of the human body, and therefore presumably on that of the deposits which it forms; and, secondly, to ascertain the influence, if any, exerted by these constituents in retarding the conversion of the sodium quadriurate contained in the fluids of the body in gout into the biurate. The experiments were conducted in the manner described in the Goulstonian Lectures already mentioned, and the vegetables employed were spinach, Brussels sprouts, potato, asparagus, savoy cabbage, French beans, lettuce, beetroot, winter cabbage, celery, turnip tops, turnip, carrot, cauliflower, seakale, and green peas. With regard to all of these, except the last-mentioned, it was found that the presence of even a minute quantity of their mineral constituents appreciably increased the solubility of the sodium biurate.

Experiments were next undertaken for the purpose of determining whether the effects produced were due to the alkalinity of the vegetable ashes, or whether they could be referred to any one saline constituent of the vegetables. The results were to show that the solvent effect of a given ash upon sodium biurate bears no relationship either to alkalinity, or, as far as could be ascertained, to composition, as tested by the proportions of potassium, sodium, calcium, magnesium, iron salts, phosphates, sulphates, or chlorides. The solvent effect of the ash of Brussels sprouts is high, while its alkalinity is low; and the solvent effect of celery is low, while its alkalinity is high. The solvent effect of the ash of spinach is high, while its proportion of potassium salts is low; and the solvent effect of the ash of turnips is low, while its proportion of potassium salts is high. The solvent effect of the ash of potato is high, while its proportion of sodium salts is low; and the solvent effect of the ash of seakale is low, while its proportion of sodium salts is high. Analogous results were obtained from other substances, with the result of showing that the solvent effect could not be attributed to any one constituent. The most exact attainable imitation of the ash of spinach was then prepared in the laboratory, and was found to exert a retarding influence on the solubility of the sodium biurate, while the natural product greatly promoted the solubility; leading Dr. Luff to the conclusion that in the natural ash there is some combination of the mineral constituents which cannot be artificially imitated, and on which the solvent effect depends. Other experiments were then undertaken, in order to determine the effects of the same vegetables in delaying the conversion of the soluble sodium quadriurate into the insoluble biurate, and in thus giving increased time for the elimination of the former in its original condition. The general result was to show that the mineral constituents of most vegetables both increase the solubility of the biurate and delay the conversion of the quadriurate, while the mineral constituents of meat diminish the solubility of the biurate, and do not delay the conversion of the quadriurate. Dr. Luff is of opinion that he has at least approached a chemical explanation of the effects of diet in producing or retarding a tendency to gout; and he gives the first place to spinach as an article of food where such a tendency has to be combatted. Brussels sprouts and French beans occupy the next places on his list, and are followed by cabbage, turnip tops, turnips, and celery.

